# Book Reviews

**Published:** 1811-08

**Authors:** 


### CRITICAL ANALYSIS

OF

### RECENT PUBLICATIONS

IN THE

### DIFFERENT BRANCHES OF PHYSIC, SURGERY, AND MEDICAL PHILOSOPHY.

---

*Observations on the Act for regulating Mad-houses, and a Correction of the Statements of the Case of Benjamin Elliot, convicted of illegally confining Mary Daintree; with Remarks addressed to the Friends of Insane Persons. By* James Parkinson. 8vo. pp. 48. sewed. London. 1811.

The case which called forth these observations of Mr. Parkinson will be in the recollection of most of our readers. He signed a certificate testifying the insanity of a Mrs. Daintree, in consequence of which she was confined in a mad-house, where she remained about three months. Three years within a month after the time of signing the certificate, Mr. Parkinson received a subpœna, and attended as a witness on the trial of the parties implicated in depriving Mrs. Daintree of her liberty. The result of the trial was, the defendant Benjamin Elliot was found guilty, and sentenced to six months imprisonment in the House of Correction in Cold-bath fields. If this verdict of the jury be correct, Mr. Parkinson must have acted interestedly, or injudiciously; he has therefore, in our opinion, very properly published the particulars of the case, which at least justifies his conduct in the affair. The minds of the jury seem to have been influenced by the direct contradiction of the defendant's principal witness, and in some degree perhaps by Mr. Parkinson not being able to swear to him, although he had afterwards no doubt of his identity. Mr. Parkinson, before he signed Mrs. Daintree's certificate, had examined her son, who declared that she was mad; on his examination in court, on oath, this young man denied that he had ever been questioned by Mr. P. respecting the state of his mother's mind, or that he had ever acknowledged that she was insane. Unfortunately Mr. Parkinson's memory would not allow him to identify this young man, when giving his evidence. As Mr. Parkinson did not sign the certificate until he was convinced of the patient's insanity from his own observation, surely this conduct of a witness should not have made any undue impression in court.

We shall now state some of the excellent remarks which this case has drawn from Mr. Parkinson.

One of the most important articles in the Act for regulating mad-houses, is that which prevents patients being received into them without an order in writing, under the hand and seal of some physician, surgeon, or apothecary. This regulation is doubtless productive of much good; there can be few individuals legally entitled to the rank of physician or surgeon, incompetent to determine upon the sanity or insanity of a patient.

"But how widely different is it with respect to some of those persons who call themselves apothecaries, and thence presume to judge respecting diseases. Their abilities have been examined by no prescribed test, nor have they received any authority to take on themselves the delicate and important task of judging of, or of practising upon, the diseases either of the body or the mind. That in this metropolis, and in many parts of the empire, there are many respectable persons, who with the designation only of apothecaries, possess every acquirement which is requisite for the successful exercise of their profession, is well known. But it is equally well known, that there is hardly a neighbourhood which is not infested with some ignorant and illiterate being, who having learned the names of many medicines, and of some diseases, seeks a livelihood by putting the lives of his neighbours at hazard, by pretending to remove the diseases with which they may happen to be afflicted."

It admits of no question, that such men are wholly unfit to decide upon the nice distinctions between eccentricity, hypochondriasis, and insanity. Independently of their ignorance, the moral character of men, who thus dare to profess an acquaintance with a difficult profession, must be suspicious. The Act indeed provides, that if the houses be situated within seven miles of London and Westminster, and within within the County of Middlesex, it shall be visited by Commissioners appointed for the purpose, *once* at least in every year, when the patients are inspected. Beyond this distance, the visitation takes place as often as the Commissioners think fit. Now supposing that a patient has been improperly confined, a considerable time may elapse before his case may be known to the Commissioners; and before his release can be effected, great violence may be done to his feelings. Mr. Parkinson has adduced many cases illustrative of his arguments, for which we refer to his sensible and judicious publication, in which he has fully established the necessity of a revision of that part of the Act which gives so vaguely the power of confinement.

---

*Communications relative to the Datura Stramonium, or Thorn-apple; as a cure or relief of Asthma: Addressed to the Editor of the Monthly Magazine Several of them never before published.* 8vo. pp. 90. Sherwood, Neely, and Jones, 1811.

This Pamphlet, as the title expresses, consists chiefly of cases in which Stramonium has been employed in the cure of asthmatic complaints. The professed motives of the Editor in collecting and publishing these communications, do him honour, because he believes in the efficacy of the remedy, and that a more general knowledge of its effects will be beneficial to mankind, whilst he can have no other interest in its success.

After the ample discussion which this subject has already received in our pages, it may suffice on the present occasion to remark, that the cases now adduced are in favour of the remedy. The objections, however, which were advanced against its introduction into general and indiscriminate practice, in the account of the plant before alluded to, in our opinion, still remain in force.* Admitting that it possesses potent qualities, and that it has occasionally proved beneficial; we contend after a fair and full examination of the question, founding our judgment upon considerable experience, that the application of Stramonium in the form of smoke, in many cases of asthma, is dangerous, in proportion as it produces its narcotic effects on the system; the cough and dyspnœa may be quieted; but apoplexy and other serious disorders have sometimes been the result.

---

* *Vide* Medical and Physical Journal, June 1, 1811.

An

An Appendix is subjoined, containing Dr. Thornton's *description of Datura Stramonium*; an extract from Storck's account of it, published at Vienna in 1762; and an extract from the Medical and Physical Journal, May 1811.*

We cannot doubt that the attention already excited in the public mind by the various reports, and advertisements, respecting Stramonium, will induce many people to have recourse to it, and that we shall have further opportunities for collecting evidence on the subject. It is difficult to obtain the particulars of the unfavourable cases where the remedy has been used. When persons quack themselves, they are extremely unwilling to publish the mischief which has been occasioned by their own folly and imprudence.

---

*The Edinburgh Medical and Surgical Journal, No.* XXVI. *April* 1811.

Art I. *Medical Report for Nottingham. By* James Clarke, M. D.

This attentive observer commences with some sensible remarks upon the influence of the seasons in modifying, and inducing disease; a subject certainly not sufficiently regarded by practitioners in general. He then notices some objections of a *former* reviewer in this Journal, to certain opinions which he had advanced on the subject of diabetes.

The *epidemic constitution*, from April 1809 to March 1810, gives us a statement of the weather, with an enumeration of the diseases that prevailed in each month. Synochus was the prevailing disease in April and May. In July, Rubeola was prevalent, and in one instance proved fatal, previous to the appearance of the eruption.

---

* The Editor of these Communications introduces the Extract from the Medical Journal with the following observations: "The Medical Journal noticed Stramonium last month (May) for the first time. The writer of the article fell however into an error, conceiving that Verax and Fisher (or *Rees*) were the same person, and detesting, as every honest man must, the imposture of the pretended Surgeon, has unwillingly given credence to the unquestionable authority of the Monthly Magazine." In reply, we may observe, that notice was taken of Fisher's Treatise on Stramonium, in the Medical Journal for Jan. 1811; further, that though it might be interpreted that the writer of the account of Stramonium suspected that Fisher and Verax were assumed titles, by the same individual, he by no means insinuated that Dr. *Rees* was implicated; we rather suppose too, that the Editor of the Communications intended to have written *Reece*.

"*October.*—Rain in the night of the 13th, 18th, 24th, and 25th. The weather this month cloudy and hazy.

"*Ophthalmia.*—This disease became very general this month, and was considered contagious. It attacked the patient very suddenly, often in the middle of the night, with a violent pain in the orbit, and a sensation of sand in the eye. On rising in the morning when the attack has been severe, the eye discharged a purulent fluid, but this was only in the more acute cases; in the majority of instances, the disease was very slight, and was removed in two or three days by a simple collyrium and a saline cathartic.

"*Pneumonia.*—Some few mild cases of this disease in the early part of this month.

"*Dysenteria* was much more frequent than usual, not without suspicion that it arose from contagion, from the soldiers on furlough who had returned from foreign service. Debility of the whole alimentary canal was a complaint in those who had suffered from dysentery or cholera.

"*Catarrhus* was prevalent, and the fever in almost every instance typhoid.

"*Pertussis and Rubeola* were seen in a few sporadic cases.

"*Typhus* was unusually frequent this month, and certainly proved very contagious; it generally passed through the family. The disease commenced with very strong rigor, often in the night, followed by heat of skin, great thirst, headache, lassitude, languor, pain in every part of the body, sometimes more violent in the chest and sides, and then attended with a cough, which induced many to have recourse to bleeding, of which they had soon cause to repent, for the patient seldom recovered a copious general bleeding; the pulse was extremely feeble, not always quick, the tongue much furred, oftentimes blackish at the root, and when put out tremulous; the bowels at the commencement costive, but as the disease advanced, without medical assistance, a diarrhœa generally followed. With some, the disease was accompanied with sore throat; the fever was not violent, seldom attended with delirium, but lingering. Nearly one-half of the out-patients of the hospital, under the care of the reporter, who had been received for other diseases, had slight attacks of typhus. The only fatal case of those under the care of the reporter, was attended with a vomiting of thick greenish and coffee-ground-coloured fluids. Medical assistance was not called to this patient until some hours after this fatal symptom had come on; the extremities had become cold, on the forehead a cold clammy sweat, and no means that were resorted to had the effect of restoring the natural heat of the body; the countenance was most ghastly, the true "facies hypocratica" was marked in each feature; and entire prostration of strength, the arms hanging down loosely by her side, the hair dishevelled, and a total insensibility to all surrounding objects. Opium, blisters, and every method that was tried proved of no relief; and in twenty-four hours from the first seizure, this fine young girl was an inanimate and unsightly corpse."

ART.

### Art. II. *Case of Apparent Paralysis of the lower Extremities removed by the patient being attacked with Hepatitis and Gout.* By Mr. Mellor, Surgeon, Stafford.

"May 23.—Mrs. H———, aged 40, full habit of body, menses regular; has been unwell a month or five weeks; complaining of general debility; great weakness and numbness of the lower extremities; has now much pain in the lower part of the back, sacrum, and pelvis; on examination of the spine, she complained of great tenderness of the sacrum and back; she cannot walk, or even stand, without being supported, nor can she turn herself in bed, or move the lower extremities. The bowels are very costive; the bladder appears to be paralytic, as she does not part with her urine oftener than once in 24 hours, and then in the quantity of two quarts; the skin is cool, tongue clean; pulse 80 and soft.

"She was ordered to take six grains of calomel immediately, and a purging mixture every four hours afterwards, composed of sulphas magnes. infus. sennæ, and pulv. jalapii. This procured several large evacuations. She was then directed to take a mixture with gum guaiacum, also to rub the spine with lin. volat. to use the warm bath every day, and to apply a blister to the sacrum.

"*May 30th.*—Complains of much pain in the epigastric and right hypochondriac regions; is completely jaundiced; has a short troublesome cough; the skin hot; tongue parched, and loaded with a yellow fur; great thirst; pulse 110, full and hard. An inflammation of a rose colour has attacked the outside of the right foot, which extends towards the toes, attended with swelling and great pain; the urine is in small quantity; makes it more frequent than she did, and it is very yellow; stools of a dirty white.

"I bled her to the quantity of sixteen ounces; put her upon the antiphlogistic plan; gave her saline medicines, with nitr. and vin. antim.; purged her smartly every day with calomel and infus of senna, and pulv. jalap. The blood was highly inflamed; she was bled again on the 31st, also on the first of June, and on the fifth.

"*June 2d.*—The gout has attacked the other foot.

"*June 4th.*—The inflammation has attacked both hands, and even extends to the fingers; the feet are better; has more feeling in her legs, and can move them a little; in other respects much the same. Ordered to continue her present plan."

From this time she recovered fast, and by the 20th of June was entirely well. Mr. Mellor concludes with enquiring, "if she had not been attacked with this violent inflammatory disease, would she have become paralytic?"

### *Case of Hæmorrhœa Petechialis, successfully treated.*

There is nothing peculiar in this case of a child seven years of age; the medicine was decoction of bark, with muriated tincture of iron: the diet, fresh animal food; ripe fruits; vegetables; half a pint of port wine, diluted with an equal quantity quantity of water, every twenty-four hours; and the body was sponged every four hours with cold vinegar and water.

*Two Cases where Bleeding, so as to produce Syncope, cured the Disease immediately, without the aid of Medicine.*

"Miss Needam, aged 18, apparently of a full habit, pale complexion, but delicate constitution, short chest, has, for the last two years, been subject to violent attacks of apasmodic asthma, and an incessant, short, and irritable cough.

"*October* 15*th.*—Was attacked after dancing with the cough, which is incessant, recurring every second of time, and which appears to arise entirely from irritation; she has no pain or difficulty in breathing; pulse soft, but frequent; she has been coughing without intermission thirty-six hours; has taken opium, æther, camphor, assafœtida, and inhaled the steam of warm water, and also the vapour of æther, but without the slightest good effect. I bled her from a large orifice to the quantity of twelve ounces, until she fainted; she was very sick and vomited a good deal. The cough immediately ceased, and did not return in the slightest degree again.

"Mrs. S——, aged 36, spare habit, had been subject to attacks of acute rheumatism; complains of violent and acute pains in her knees, ankles, elbows, and wrists; is unable to walk without the support of two persons; skin cool; tongue rather white; pulse 80, but full, and rather hard; urine high-coloured; joints not swelled or inflamed.

"I bled her from a large orifice to the quantity of sixteen ounces; she fainted, and vomited, and was near an hour in recovering. I called upon her the next morning; found her busy in her domestic affairs; said she had had a very good night; was entirely free from pain, and could walk as well as usual, and has had no return of her disease."

---

ART. III. 1. *Case in which the Larvæ of an Insect were voided in the Urine.* 2. *Cases of Poisoning by Foxglove and Corrosive Sublimate.* 3. *Remarks on Enlargement of the Heart.* In a letter to Dr. Duncan jun. from William Henry, M. D. F. R. S. &c.

The subject of the first of these cases frequently voided gravel, and had strong symptoms of stone in the bladder, and once actually passed a small calculus.

For five or six weeks, there were discharged along with the urine, "the larvæ of an insect pretty exactly resembling the common maggot. They are not only alive but vivacious, and, besides those which are entire, the heads and bodies of several others may be observed, detached from each other. Of the entire insects he has frequently discharged three or four at once; and they appear in the urine, when it is perfectly free free from sand, and even when it is received into a glass vessel."

The insect has a hard crustaceous head, with long maxillæ, like those of coleopterous larvæ. The body appears to have been soft and pulpy, and when magnified resembles *curculio nucum*. A semi-transparent line is seen running from the head to the tail. The body is covered with short hairs or *setæ*. The colour is dirty yellowish; head bright chocolate.

### *Effects of an excessive dose of Foxglove.*

On the 17th of October, 1809, Dr. Henry was called to visit Alice Grice, aged about 60, as a home patient of the Manchester Infirmary. She had laboured under ascites for some months, and though already an out-patient of the charity, had taken, at the persuasion of a neighbour, a strong decoction of foxglove, prepared by boiling two handfuls of the leaves in a quart of water during half an hour. Of this, about seven o'clock on the morning of Sunday the 15th, she drank about ten ounces; in less than an hour she began to be sick and discharged part of the contents of the stomach.

" Enough, however, was retained to excite violent retching and vomiting throughout the whole of that and the following day, during which, every thing that was taken was instantly rejected. In the intervals of sickness she was excessively faint, and her skin was covered with a cold sweat. The tongue and lips swelled, and there was a constant flow of viscid saliva from the mouth. Very little urine was voided on Sunday; and, on the two following days, the action of the kidneys was entirely suspended. When I saw her on Tuesday the sickness had somewhat abated, though it was still extremely distressing. The tongue was covered with a white fur: the ptyalism continued, though in a less degree; and the breath was fœtid. The pulse was low, irregular, (not exceeding forty) and after every third or fourth pulsation an intermission occurred for some seconds. She complained also of general pains in the limbs, and of cramps in the legs.

" Though the danger appeared to me to be greatly diminished, yet something was absolutely necessary to abate the harassing sickness. I directed, therefore, effervescing draughts, prepared with infusion of columbo and carbonate of ammonia, with the addition of ten drops of laudanum to each, to be taken every three hours. In the intervals, thirty drops of a mixture of æther and the compound spirit of ammonia, were given occasionally; and she was supplied freely with wine from the hospital. Under this treatment the sickness and vomiting soon abated, and she gradually returned to her former state of imperfect health. The pulse, however, did not completely regain its regularity before the commencement of the following week."

" The case of poisoning by corrosive sublimate, " Dr. Henry observes, " is chiefly interesting from its bearing upon a point of some importance, which was discussed on a trial for murder at the Lancaster assizes in 1808. The defendant, on that occasion, appeared to owe his his acquittal to the fact, that no poisonous substance could be detected after death, in the contents of the stomach or bowels, though the proper tests were applied with great skill and judgment, by Dr. Bostock. It was the declared opinion, however, of that physician at the time, and was afterwards proved by a course of experiments on animals, that a poison may produce fatal effects, and yet be so completely evacuated by vomiting or purging, as to leave no trace discoverable by chemical analysis, in the contents of the alimentary canal. Some time after this period, the following case occurred to my friend Dr. Holme, who obligingly pointed it out to my notice.

"Hannah Tomlinson, aged about 20, was induced by a series of ill-treatment and by the apprehension of pregnancy, to form the resolution of destroying herself. With this view, she poured about a quarter of a pint of hot water on an ounce of corrosive sublimate, and drank the whole of what the water could hold, both dissolved and in suspension. The act of swallowing was attended with a violent spasm of the glottis, and a small quantity of the liquid was rejected from the mouth. In less than half an hour she became extremely sick, and discharged the contents of her stomach. The retching, however, continued, and she threw up a considerable quantity of blood. On the following day the sickness had not abated, though the hæmorrhage had ceased. From this time to the period of her death, which, notwithstanding the most judicious treatment, happened on the sixth day, she continued to labour under sickness, anxiety, restlessness, quick pulse, and universal pains in the limbs. To these symptoms supervened, on the fourth day, great pain about the scrobiculus cordis, and tenderness on pressure; and a few hours before death, a complete paralysis of the upper and lower extremities took place.

"Some of the fluid which had been vomited about twelve hours after she had taken the sublimate was carefully examined by Dr. Roget (now physician in London) and myself. The tests applied were those which are described in my *Elements of Experimental Chemistry*, Vol. ii. page 393; but neither in this liquid, nor in that found in the stomach after death, were any traces of the poison discoverable.

"On the day after her decease, the body was opened by Dr. Roget, in the absence of Dr Holme from town. The external appearance of the stomach and intestines was perfectly natural. About two ounces of a thick yellow ropy fluid were found in the stomach, which was but moderately distended with air. On its inner surface, numerous dark red spots, indicating inflammation of the villous coat, were observable. They extended the whole length of the smaller curvature, and occupied the greater part of the fundus, but did not appear in the lower portion of the large curvature. To me they seemed to resemble, very closely, a similar appearance which I have three or four times observed in the stomachs of persons who have died of hydrophobia. No abrasion of the villous coat was perceptible. The inner coat of the duodenum, as far as the middle of its length, presented the same appearance of inflammation. The lower part of the œsophagus, for about three inches above the cardia, was slightly inflamed, but higher up it was of a natural colour. The liver and spleen were sound; the gall bladder more empty than usual. The left kidney was of a looser texture than natural, and a small a small abscess was discovered in it filled with pus. The bladder was empty, and exceedingly contracted*. The uterus was of the natural size, and its cavity exhibited no marks of pregnancy. The ovaria were somewhat enlarged, and the left contained several hydatids; but no corpus luteum could be detected in either of them. The heart and lungs, it may be added, were perfectly sound."

### *Enlargement of the Heart.*

"CASE 1.—Thomas Leech, aged 20, late a sailorin the Royal Navy, had laboured for nearly three years under palpitation of the heart and shortness of breathing. About two years before, he had been induced, by the urgency of his complaints, to desert from his ship, but had been taken, and sentenced to undergo the punishment of the fleet. His complaints soon afterwards increased so rapidly, that in a short time he received his discharge from service. When I first visited him, on the 23d of April, 1809, as a home-patient of the Manchester Infirmary, his situation was truly deplorable. The pulsation of the heart was felt as low as between the ninth and tenth ribs; was extremely violent, and was attended with that peculiar jarring, which has been described by Dr. Ferriar, and other medical writers. The pulse at the wrist was 128, small and indistinct; the breathing laborious; and every attempt to lie down brought on a sense of suffocation. He was obliged, therefore, to sit with his head reclined upon his arms, which were supported by a table. The belly was considerably swelled; the legs œdematous; and the urine scanty and high-coloured. He had a constant tendency to sickness, and frequently severe pain in the abdomen. His dissolution, indeed, seemed to me so near at hand, that I attempted nothing but to palliate the most urgent symptoms. On the 1st of May he died; and with great difficulty I obtained permission to inspect the body, which was done, under circumstances of haste and interruption from the young man's friends, by Mr. Ransome, one of the surgeons to the Infirmary."

2. William Barlow, aged 16, was seized suddenly with violent pain in the head, and lost the use of the right side. He soon recovered this, however, but was attacked with still more distressing symptoms. The belly and legs began to swell, the breathing became short and laborious. The œdema soon extended to the face, the eyelids, and the scrotum. He had violent cough and spitting; the lips had a livid hue. The palpitation of the heart was considerable, and it appeared to be situated lower than natural; the stroke was indistinct and distant. There was a visible pulsation of the veins of the neck. At the wrist the pulse was small, irregular, rapid, and not always synchronous with the motions of the heart.

---

* No urine was voided after the third day, and on introducing the catheter repeatedly, the bladder was found empty. Suppression of urine (which took place also in Grice, from an over-dose of one of our most active diuretics), appears to be no uncommon effect of the administration of poisons. Vide Haller, Elem. vii. 396, edit. Lausan, 1778.

The

The appetite was tolerably good; the bowels regular, and the urine natural in quantity.

He died on the 14th of January. "On the following day, the body was opened by Mr. Ransome. The right sac of the pleura contained about a pint and a half of serum; and about nine ounces were found in the pericardium, which had no adhesion to the heart. The heart itself was enlarged, and the parietes of both ventricles were thicker than natural. In the left ventricle, the *columnæ carnæ* were in some places cartilaginous, and in others partially ossified. The right ventricle contained one of those substances which have been erroneously called polypi. But the chief seat of disease was in the right auricle and sinus venosus, which were dilated to twice their natural size, and were ossified in spots. All the valves of the heart, and those at the origin of the large arteries, were healthy. The weight of the heart was 14 troy ounces, perhaps double what it ought to have been in proportion to the size of the subject.* In the abdomen, the only deviation from a sound state of the viscera was an enlargement of the right kidney.

"The pain of the head and paralysis (though not the subsequent removal of the latter), were satisfactorily accounted for on examining the brain. Its membranes were sound, and no hydropic effusion had taken place between them. But in the posterior part of the brain, immediately above the *cornu ammonis* of the left lateral ventricle, an abscess was discovered, containing about two drachms of dark-coloured pus. The ventricles themselves contained, if any thing was remarkable, less than their natural quantity of fluid."

---

### Art. IV. *Case of Deformity of the Face and Throat from Burning, removed by Chirurgical Operations.* By George Nesse Hill, Surgeon, Chester.

A boy, eight years of age, was severely burned from the breast bone along the throat, chin, under lip, and left side of the face to the eye. In about nine months the surface of the burned part was cicatrized; and during this process, the integuments, from the upper edge of the sternum to the chin, were formed into a hard ridgy band, near two inches wide, and which every day brought the lower jaw nearer to the breast, so as wholly to preclude the possibility of approximating the inferior to the upper lip; occasioning considerable impediment to speech and a constant dripping of saliva; a dragging of the whole cheek downwards and backwards to the left ear, which was shrivelled. The mischief was augmented by a complete eversion of the inferior eye-lid; and so firm were the parts beneath it become, that the eye-lash lay fixed nearly flat on the cheek, the inner membrane forming, from the shape and unyielding structure of the tarsus, an

* Vide Senac, Traité du Cœur, i. 131.

arched arched fold, tightly embracing the inferior portion of the globe, and leaving it constantly exposed.

Under these circumstances it was determined, in consultation, to divide, freely, as many of the constricted masses of integument and subjacent parts as would admit of such an operation, and effect the intentions indicated. This Mr. Hill executed in the following manner.

"The hard band which reached from the chin to the sternum, being relaxed as much as possible, by bringing the head forwards and downwards, a large needle, having a considerable curve and cutting edges, was passed through the (comparatively) soft parts behind it, close to the trachea, from one side to the other, including as much lateral skin as possible. Upon this instrument, held firm, the burnt mass was with considerable difficulty divided by a scalpel; the needle being thus freed, a large vein and two arteries which it had cut were taken up, and the farther division of the skin, cellular membrane, and muscular fibres completed laterally, as far as was practicably safe. Thus the stricture was so effectually removed, that our little patient could shut his mouth again with ease; the left angle was also greatly relieved, and the eyelid visibly amended; but so painful and tedious had this operation been, and the loss of blood so considerable, that it was deemed prudent to decline attempting more that day. The wound was dressed with a common dressing, and the head fixed as far backwards as possible, resting against a firm bolstered splint, which reached from the occiput to the coccyx; but in a few days, this method being found unsteady and incapable of admitting the head to be in every situation retained in that position, I contrived a machine from the girls' steel collar, which effectually answered. The wound healed smoothly in seven weeks, all contraction being removed, and but little hardness remaining. The head now resumed its natural position, and the thickened hard lip recovered its true situation, having become much softer. Notwithstanding which favourable change, the machine was still worn, night and day, without inconvenience, in order to keep the muscles and integuments of the throat as much as practicable upon the stretch. The folding or tucking inwards of the mouth, with the depressed and everted eyelid, now became the objects of further proceedings. Accordingly, the head being firmly fixed in the machine, and the heroic boy seated on the knee of an assistant, an incision was made upon the forefinger of the left hand, previously introduced into the mouth, reaching from near the angle of the lips to within a small distance of the ear, through a very hard, almost insensible, mass of scorched integuments, and carried downwards as deep as the parts would admit without dividing the internal membrane of the cheek: thus the puckering or folding inwards was removed, and a space gained sufficient to restore the injured eyelid about half way towards its true place. But in consequence of its long-continued displacement, the membrane had become greatly elongated, and now formed a large fold, impeding perfect restoration. To remedy this defect, having a tenaculum in my hand, I pushed it through a semicircular fold of the superfluous part, which, by one stroke of a pair of scissars, was cut away, the lids now closing nearly as well as those of the the uninjured organ, independent of any efforts of the patient for that purpose. Prepared sponge being inserted into the wound of the cheek, was suffered to remain there till ejected by suppuration :' it cicatrized in about three weeks. To the eye a cold saturnine cataplasm was applied : it healed without trouble. But in defiance of all care, and the unremitted assistance of compresses, adhesive plasters, and bandage, the cheek was no sooner well than a dragging downwards of the lid became every day more evident, which pulled it into an arch or cup-like form ; here the tears accumulated and ran over. My little sufferer viewing himself one day in a glass, pointed out where he felt the dragging or tightness at the side of the nose, and a less degree beneath the external canthus. Consenting to have it removed, I took a knife and made a semilunar incision from the nose towards the temple, nearly down to the bone, and filled the wound with sponge tent. The eye was now completely closed, and covered with folded linen, wetted with cold saturnine solution. Upon the sponge coming away, the eyelid was retained by slips of adhesive plaster, carried up to the forehead. In three weeks this third incision was healed ; the relaxed parts had greatly recovered their tone ; the tears ceased to flow down the cheek."

In the treatment of burns on every part of the frame, but especially about the face and hands, the great object is to prevent those unseemly and incapacitating contractions so apt to occur. With a view to enforce this principle, we insert the following case, given by Mr. Hill, and which is properly contrasted with the preceding.

" Since transcribing the foregoing narrative from my notes, a case of extensive burn of all the right side of the throat, neck, and shoulder, has fallen under my care ; the subject a fine, tall, thin girl, 11 years old, and it happened in consequence of setting fire to her tippet when alone up stairs. This garment being tied behind, she could not disembarrass herself from it without coming down for assistance. Cold water was liberally applied before my arrival, I found her quite easy, and directed a continuance of the water, during the preparation of a quantity of the potatoe cataplasm sufficient to cover the whole of the burnt parts. During the first 48 hours after the accident, these were occasionally renewed, and kept constantly cool by means of a sponge, imbued with the drainage liquor from the grated roots ; subsequently, a dressing of cerate, softened with sp. tereb. spread on thin lint and covered by fourfold linen wetted with liq. plumb. procured an easy free digestion. But as the mischief was extensive, and the mastoid muscle much injured, I was very anxious to prevent contraction and wry neck. Accordingly, I fixed the head in a similar machine to that already described, so as to preserve the chin in a state of constant elevation ; by which method impending disfigurement was completely avoided, and smooth cicatrization obtained in ten weeks.

---

Art. V. *Account of the good effects from inhaling the Smoke of several species of Datura, especially the Datura Fastuosa, in Asthma. By* Thomas Christie, M. D.

This account is not very accurate; the several species enumerated, merated in the head of the article, in the body of it, appear to be only two. The author has heard of the relief obtained from smoking the Datura Stramonium in spasmodic asthma; and acquaints us that he has employed the smoke of the *datura* in a variety of cases of asthma, and that it always procured relief when used before the accession of the paroxysm, or even after its commencement; but he is sorry to add, "that it did not often prevent the repetition of the fit, unless great attention was at the same time paid to diet and regimen."

"Its immediate effects were a sense of heat in the chest, followed by expectoration, and attended frequently with temporary vertigo, or drowsiness, and sometimes nausea."

---

Art. X. *A Case of Rupture of the Lungs in Parturition.* *By* Mr. W. Balfour, Surgeon, Edinburgh.

During labour, a woman, 25 years of age, had made extravagant voluntary exertion for the propulsion of the fœtus. In these efforts, it appeared that some part of the lungs had given way, and when Mr. Balfour first saw her, the face was tumid. After delivery this tumefaction subsided, but is stated soon to have returned.

"The countenance was now, within half an hour after the separation of the placenta, much swollen, but especially the upper eyelids. The patient pointing to the bronchia, complained of a sense of suffocation, of swelling about the neck, and soreness in the right side of the thorax, toward the upper and back part. A crepitus was distinctly felt in the right arm, and the shoulder, neck, and face, exhibited the same unequivocal symptoms of emphysema. The upper eyelids pressed so hard upon the eyes as to occasion pain. Six or eight punctures were made in them, from which, in a short time, so much air was extricated, that the patient declared she could look up. In a few days the patient recovered."

A similar case has occurred in our own practice. A young and healthy woman in her first labour, made great voluntary exertion, which was followed by emphysematous tumour of the face, to a degree that completely obscured the features. It extended in a smaller degree to the neck and shoulders. In twenty-four hours this subsided without assistance from art. In this instance there was no pain in any part of the lungs, indicatory of a point where a rupture had taken place.

---

Art. XII. *Case of Suppression of Urine, from Inflammation of the Neck of the Bladder, brought on by the improper use of Injections, and cured by the use of Camphor.* *By* Mr. Hamilton Baillie, Surgeon, R. N.

This alarming case succeeded to a gonorrhœa which appeared on the 20th of January. On the 23d the patient, a plethoric sailor 21 years of age, used an injection of a weak solution of sulphate of lime. On the 28th a suppression of urine took place. At four o'clock in the morning of the 1st of February delirium came on, the pulse was very feeble, and quick ; and some paralysis of the lower extremities. As it was judged that the inflammation about the neck of the bladder had subsided, it was hoped that the catheter might be passed into the bladder ; but from the distress occasioned by the former unavailing attempts, the patient refused to submit to the trial. In this extremity, it was proposed by Mr. T. King, assistant surgeon, to employ camphor ; which was given in doses of ten grains every hour. A few minutes after the patient had taken the third dose, an involuntary discharge of urine took place, the pulse becoming fuller and slower : and by the time he had taken the fifth dose, several pints of urine, the greater part discharged involuntarily, were voided. The camphor was then discontinued, and in a few hours every unpleasant symptom disappeared.

---

Art. XIII. *History of three Persons who were nearly suffocated, and of one who perished, from the irrespirable Gases arising from a Coal Fire. By* David King, M. D.

The subjects of the symptoms described by Dr. King were sailors, who were affected in consequence of a fire having been kindled in the hold of their vessel, and their neglecting to leave the hatches open. The cases are well related, but will not admit of being abridged.

---

Hortus Elginensis; *or a Catalogue of Plants, indigenous and exotic, cultivated in the Elgin Botanic Garden in the vicinity of New-York.* By David Hosack, M. D. Prof. of Botany and Materia Medica in Columbia College, Member of the American Philosophical Society, &c. 2d Edit. 8vo. New-York. 1811. Plate. pp. 65.

*A Statement of Facts relative to the Establishment and Progress of the Elgin Botanic Garden, and subsequent Disposal of the same to the State of New-York.* By David Hosack, M. D. &c. &c. &c. 8vo. New-York. 1811. pp. 56.

The cultivation of plants, and the construction of a garden, seem so congenial to the nature of man, that no surprize is felt on observing him, from the earliest period of his existence istence through all subsequent times, applying to this delightful avocation. From the first garden, the terrestrial Paradise, where

> "Universal Pan,
> "Knit with the Graces and the Hours in dance,
> "Led on the eternal Spring,"

tradition or history has afforded details of this art. If it cannot be proved it can hardly be admitted, that the gardens of the Hesperides, of Adonis, and Alcinous, flourished no where but in song: or that Solomon, who was so well acquainted with all vegetables, possessed no other garden but that which was planted by Cotovicus in the *Itinerarium Hierosolymitanum.* If these, indeed, were the visions of the muse, or the fabulous creation of faithless historians, authentic records yet remain of cultivated collections of plants, existing at a very early period, and bearing the semblance of infant science. Attalus the 3d, and the last king of Pergamus, was remarkable for having fled from the cares of government to cultivate a garden, 140 years before the Christian æra. In this garden he grew many poisonous plants, for the purpose of making experiments on criminals, with a view to ascertain the properties and powers of counter-poisons. Both Pliny and Galen speak of a Greek named Castor, who had a garden at Rome, in which, when he was an hundred years old, he demonstrated plants, and taught his pupils to distinguish rare and useful species. When learning revived from its long and gothic torpor, the study of Botany was among the earliest efforts of intellect: and the collection, culture, and arrangement of plants, was one of the first of the means employed to restore, or to create, this science. A botanic garden, formed at Padua in 1533, is intitled to the honourable circumstance of being the first public institution of this kind in modern Europe. Lucas Ghinus, a botanical physician, called by Matthiolus another Dioscorides, was not only an early promoter, but the first Professor of Botany in Europe, and taught the science in the schools of Bologna from 1527 to 1555; where by the force of his arguments and the influence of his reputation, he procured a public garden to be established in 1547. This, which followed that of Padua so closely, was, probably, the second establishment of the kind. As the mind became more enlightened in the study of nature, the utility of these collections, where the growth, the habits, and changes in vegetable life might, at leisure, be observed, became still more obvious. Individuals, as well as Colleges and Universities, rapidly multiplied this method of studying Botany,

 which which connected with a prevailing propensity of the human mind, the means of developing the truths of nature. Paris, Oxford, Cambridge, Leyden, Upsal, &c. soon had public gardens :—Gesner constructed one at Zurich, Clifford in Holland, and Sherard in England, with a spirit of munificence which the historian of science will not fail to record, promoted this great object. The garden at Chelsea, given to the Apothecaries Company of London, by Sir Hans Sloane; and that of the Tradescant's, which once existed in South Lambeth, in a spot where yet, perhaps, "many a garden flower grows wild," press upon the attention :—but we hasten to the subject of our Analysis.

"The establishment of a Botanic Garden in the United States, as a repository of the native plants of this country, and as subservient to the purposes of medicine, agriculture, and the arts, is doubtless an object of great importance. Impressed with the advantages to be derived from an institution of this nature, I have anxiously endeavoured, says Dr. Hosack, ever since my appointment to the Professorship of Botany and Materia Medica in Columbia College, to accomplish its establishment. Disappointed, however, in my first applications to the legislature of the State, soliciting their assistance in so expensive and arduous an undertaking, I resolved to devote my own private funds to the prosecution of this object, trusting that when the nature of the institution should be better and more generally known, and its utility fully ascertained, it would receive the patronage and support of the public.

"Accordingly, in the year 1801, I purchased of the Corporation of the City of New York twenty acres of ground, situated on the middle road between Bloomingdale and Kingsbridge, and distant from the city about three miles and a half. The view from the most elevated part is variegated and extensive, and the soil itself of that diversified nature, as to be particularly well adapted to the cultivation of a great variety of vegetable productions. The greater part of the ground is at present in a state of promising cultivation, arranged in a manner the best adapted to the different kinds of vegetables, and planted agreeably to the most approved style of ornamental gardening Since that time, an extensive conservatory for the more hardy green-house plants, and two spacious hot houses for the preservation of those which require a greater degree of heat, the whole exhibiting a front of one hundred and eighty feet, have been erected, and which, experience has shewn, are well calculated for the purpose for which they were designed. The whole establishment is surrounded by a belt of forest trees and shrubs, both native and exotic, and these again are enclosed by a stone wall two and a half feet in thickness, and seven feet in height.

"As it has always been a primary object of attention to collect and cultivate in this establishment the native plants of this country, especially such as are possessed of medicinal properties, or are otherwise useful, such gardeners as were particularly acquainted with our indigenous productions, have been employed to procure them."

This is, shortly, the history of founding the Elgin Botanic Garden. In 1806 the collection of plants in it had arrived at a point when it was deemed expedient to print a catalogue of them.

"Since that time the Institution has been greatly improved, and by an act of the legislature passed on the 12th day of March, 1810, has been purchased by the State for the benefit of the Medical Schools of New York. It will also be perceived by a comparison of the present with the former edition of the *Hortus Elginensis*, that very considerable additions have been made to the collection both of the foreign and indigenous plants contained in that establishment. Gratitude demands of me on this occasion, Dr. Hosack observes, an acknowledgment of the obligations I am under to many distinguished botanists, both abroad and at home, who have contributed to this Institution. In this number are to be enumerated my much esteemed friend and instructor, *Dr. James Edward Smith*, the learned President of the Linnæan Society of London; the late Professors *Vahl*, and Mr. *Hoffman Bang*, of Copenhagen; Mons. *Desfontaines* and *Thouin*, the celebrated Professors of botany and agriculture of the Medical Schools of Paris; *Mr. Salisbury*, proprietor of the Botanic Garden at Brompton, near London; the late *Dr. Fabroni*, Director of the Royal Museum of Florence; *Dr. Bostock*, the learned President of the Botanic Institution of Liverpool; *Dr. Lettsom*, of London; *Dr. Andrew Michaux*, editor of the Flora Boreali Americana, and author of the very valuable History of the Forest Trees of North America, now published at Paris; my much esteemed friend *Dr. Alire Raffineau Delile*, of the Institute of Egypt; *Dr. Alexander Anderson*, Superintendant of the Botanic Garden at St. Vincents; and *Baron De Schack*, of Martinique. From these gentlemen I have received many rare botanical works, and some of the most valuable plants in this collection."

Though the collection in the Elgin Garden is not so large as in some older establishments in Europe, it is respectable both for number and quality. Of the indigenous plants of America we notice 1215 species: among these upwards of 200 are employed in medicine. Of plants possessing medicinal properties this seems a great number, but many of them possibly derive their title from popular opinion only; but even this title, as founded on a species of experience, is not to be slighted. Some of them have an established reputation: cinchona, ipecacuanha, jalapium, &c. are instances. It is a curious fact in the history of Medical Botany, that when Europe remained in utter darkness on this subject, the Mexicans had appropriated a considerable space of ground, near their capital, to the sole purpose of rearing the indigenous medicinal plants. About 1515 the Spaniards found this garden in a high state of cultivation. It is impossible to say how long it had existed prior to that date, but probably it had been the work of some generations.

In running over the "Statement of Facts relative to the Establishment tablishment and Progress of the Elgin Botanic Garden," an opportunity is afforded of contrasting the love for science, and the ardent perseverance of Dr. Hosack, with the neglect and frigid procrastination of the Legislature of the State of New-York. The liberality and candour of the individual is finely opposed to the cold, calculating, trading spirit of the public body. After a higgling delay of some years, the Garden with its plants, stoves, and conservatories, of which a pleasing view is given in the *Hortus*, has been conveyed to the State.

No region of the earth seems more appropriate to the improvement of Botany, by the collecting and cultivating of plants, than that where the Elgin Garden is seated. Nearly midway between the northern and southern extremities of the vast American continent, and not more than 40 degrees to the north of the equator, it commands resources of incalculable extent; and the European Botanist will look to it for additions to his catalogue of the highest interest. The indigenous Botany of America possesses most important qualities, and to that, we trust, Prof. Hosack, the projector, and indeed, the creator of this Garden, will particularly turn his attention. It can hardly be considered as an act of the imagination, so far does what has already been discovered countenance the most sanguine expectations, to conjecture, that in the unexplored wilderness of mountain, forest, and marsh, which composes so much of the western world, lie hidden plants of extraordinary forms and potent qualities.

From the scientific spirit and persevering industry of Dr. Hosack, every thing may be augured. Already has he projected an American Botany, or a *Flora of the United States*, to be illustrated with coloured Plates, similar to those in the "*English Botany*" of our ingenious countryman, Dr. Smith. Considerable progress, we are informed, has already been made in obtaining materials for this work; but we regret that its completion depends on a contingency—the permanent preservation of the Elgin Botanic Garden. In the madness of political contention, in the apathy with which governments contemplate the advance of science, in the illiberal finesse and the low juggling of party, we may look for the occasional destruction or suspension of every rational project; but we hope these accidents will not frustrate the enlarged and enlightened intention of Dr. Hosack, but rather induce him to extend his *Flora*, and make the whole of the American continent his Garden.

MEDICAL

